# Supplementary material for: High Antimicrobial Susceptibility of Cloacal Enterococci and Escherichia coli from Free-Living Dalmatian and Great White Pelicans with Detection of Cefotaximase CTX-M-15 Producing Escherichia coli ST69
Source: Antibiotics (Basel). 2025 Jan 14;14(1):83. doi: 10.3390/antibiotics14010083 (PMC11761880; doi:10.3390/antibiotics14010083)
Supplement: Supplementary file 1 [file antibiotics-14-00083-s001.zip › antibiotics-3326558-supplementary.pdf]

High Antimicrobial Susceptibility of Cloacal Enterococci and *Escherichia coli* from Free-Living Dalmatian and Great White Pelicans with Detection of Cefotaximase CTX-M-15 Producing *Escherichia coli* ST69

Supplementary Table S1. Primers and protocols employed in the PCR analyses to detect genes encoding for resistance to different antibiotics selected based on resistant phenotype.

Gen	Primer sequences (5′→ 3′)	Thermal cycling conditions			[Reference] (Amplicon size)
*bla*CTX-M-1	F: GTTACAATGTGTGAGAAGCAG	*94ºC*	7 min.	1 cycle	
	R: CCGTTTCCGCTATTACAAAC	*94ºC*	50 sec.		[52]
		50ºC	40 sec.	35 cycles	(1017 bp)
		68ºC	1 min.		
		68ºC	5 min.	1 cycle	
*bla*CTX-M-9	F: GTGACAAAGAGAGTGCAACGG	*94ºC*	3 min.	1 cycle	
	R: ATGATTCTCGCCGCTGAAGCC	*94ºC*	45 sec.		[53]
		62ºC	45 sec.	30 cycles	(857 bp)
		72ºC	45 sec.		
		72ºC	10 min.	1 cycle	
*bla*SHV	F: CACTCAAGGATGTATTGTG	*96ºC*	15 sec.	1 cycle	
	R: TTAGCGTTGCCAGTGCTCG	*96ºC*	15 sec.		[54]
		52ºC	15 sec.	30 cycles	(885 bp)
		72ºC	2 min.		
		72ºC	3 min.	1 cycle	
*bla*TEM	F: ATTCTTGAAGACGAAAGGGC	*94ºC*	3 min.	1 cycle	
	R: ACGCTCAGTGGAACGAAAAC	*94ºC*	1 min.		[55]
		60ºC	1 min.	30 cycles	(1150 bp)
		72ºC	1 min.		
		72ºC	10 min.	1 cycle	
*bla*OXA	F: ACACAATACATATCAACTTCGC	*96ºC*	5 min.	1 cycle	
	R: AGTGTGTTTAGAATGGTGATC	*96ºC*	1 min.		[56]
		61ºC	1 min.	35 cycles	(813 bp)
		72ºC	2 min.		
		72ºC	10 min.	1 cycle	
tet(A)	F: GTAATTCTGAGCACTGTCGC	*95ºC*	5 min.	1 cycle	
	R: CTGCCTGGACAACATTGCTT	*95ºC*	30 sec.	23 cycles	[57]
		62ºC	30 sec.		(937 bp)

		$72^{0}C$	45 sec.		
		$72^{0}C$	7 min.	1 cycle	

Supplementary Table S1. *Cont*

Gen	Primer sequences (5′→ 3′)	Thermal cycling conditions			[Reference] (Amplicon size)
tet(B)	F: CTCAGTATTCCAAGCCTTTG	*$95^{0}C$*	5 min.	1 cycle	
	R: CTAAGCACTTGTCTCCTGTT	*$95^{0}C$*	30 sec.		[57]
		$57^{0}C$	30 sec.	25 cycles	(416 bp)
		$72^{0}C$	20 sec.		
		$72^{0}C$	7 min.	1 cycle	
tet(K)	F: TTAGGTGAAGGGTTAGGTCC	*$94^{0}C$*	1 min.	1 cycle	
	R: GCAAACTCATTCCAGAAGCA	*$94^{0}C$*	1 min.		[58]
		$55^{0}C$	2 min.	30 cycles	(697 bp)
		$72^{0}C$	2 min.		
		$72^{0}C$	10 min.	1 cycle	
tet(M)	F: GTTAAATAGTGTTCTTGGAG	*$94^{0}C$*	1 min.	1 cycle	
	R: CTAAGATATGGCTCTAACAA	*$94^{0}C$*	1 min.		[58]
		$55^{0}C$	2 min.	30 cycles	(576 bp)
		$72^{0}C$	2 min.		
		$72^{0}C$	10 min.	1 cycle	
tet(L)	F: CATTTGGTCTTATTGGATCG	*$94^{0}C$*	1 min.	1 cycle	
	R: ATTACACTTCCGATTTCGG	*$94^{0}C$*	1 min.		[58]
		$50^{0}C$	1 min.	30 cycles	(456 bp)
		$72^{0}C$	1 min.		
		$72^{0}C$	10 min.	1 cycle	
str	F: TATTGCTCTCGAGGGTTC	*$94^{0}C$*	3 min.	1 cycle	
	R: CTTTCTATATCCATTCATCTC	*$94^{0}C$*	1 min.		[59]
		$50^{0}C$	1 min.	30 cycles	(646bp)
		$72^{0}C$	1 min.		
		$72^{0}C$	5 min.	1 cycle	

Supplementary Table S2. Primers and protocols employed in the PCR analyses to detect MLST genes.

Gen	Primer sequences (5′→ 3′)	Tª annealing (ºC)[1]	Amplicon size (bp)
adk	F: ATTCTGCTTGGCGCTCCGGG	54	583
	R: CCGTCAACTTTCGCGTATTT		
fumC	F: TCACAGGTCGCCAGCGCTTC	54	806
	R: GTACGCAGCGAAAAAGATTC		
icd	F: ATGGAAAGTAAAGTAGTTGTTCCGGCACA	54	878
	R: GGACGCAGCAGGATCTGTT		
purA	F: CGCGCTGATGAAAGAGATGA	54	816
	R: CATACGGTAAGCCACGCAGA		
gyrB	F: TCGGCGACACGGATGACGGC	60	911
	R: ATCAGGCCTTCACGCGCATC		
recA	F: CGCATTCGCTTTACCCTGACC	60	780
	R: TCGTCGAAATCTACGGACCGGA		
mdh	F: ATGAAAGTCGCAGTCCTCGGCGCTGCTGGCGG	60	932
	R: TTAACGAACTCCTGCCCCAGAGCGATATCTTTCTT		

[1]Initial denaturation (95ºC, 3 min.); 30 cycles (95ºC, 1min., Tª Annealing, 1 min., 72ºC, 2 min) and elongation (72ºC, 5min.) [60].
